# Pathway-based analyses of gene expression profiles at low doses of ionizing radiation

**DOI:** 10.3389/fbinf.2024.1280971

**Published:** 2024-05-14

**Authors:** Xihaier Luo, Seyednami Niyakan, Patrick Johnstone, Sean McCorkle, Gilchan Park, Vanessa López-Marrero, Shinjae Yoo, Edward R. Dougherty, Xiaoning Qian, Francis J. Alexander, Shantenu Jha, Byung-Jun Yoon

**Affiliations:** ^1^ Computational Science Initiative, Brookhaven National Laboratory, Upton, NY, United States; ^2^ Department of Electrical and Computer Engineering, Texas A&M University, College Station, TX, United States; ^3^ Argonne National Laboratory, Lemont, IL, United States; ^4^ Department of Electrical and Computer Engineering, Rutgers University, New Brunswick, NJ, United States

**Keywords:** gene expression analysis, radiation biology, low-dose radiation response, pathway analysis, vega

## Abstract

Radiation exposure poses a significant threat to human health. Emerging research indicates that even low-dose radiation once believed to be safe, may have harmful effects. This perception has spurred a growing interest in investigating the potential risks associated with low-dose radiation exposure across various scenarios. To comprehensively explore the health consequences of low-dose radiation, our study employs a robust statistical framework that examines whether specific groups of genes, belonging to known pathways, exhibit coordinated expression patterns that align with the radiation levels. Notably, our findings reveal the existence of intricate yet consistent signatures that reflect the molecular response to radiation exposure, distinguishing between low-dose and high-dose radiation. Moreover, we leverage a pathway-constrained variational autoencoder to capture the nonlinear interactions within gene expression data. By comparing these two analytical approaches, our study aims to gain valuable insights into the impact of low-dose radiation on gene expression patterns, identify pathways that are differentially affected, and harness the potential of machine learning to uncover hidden activity within biological networks. This comparative analysis contributes to a deeper understanding of the molecular consequences of low-dose radiation exposure.

## 1 Introduction

Radiation exposure is a critical concern with profound implications for human health and safety. While extensive research has been dedicated to understanding the effects of high-dose radiation, there is a growing recognition that low-dose radiation, even at levels previously deemed safe, may have adverse health impacts. This perception has sparked significant interest in investigating the potential risks associated with low-dose radiation exposure across various settings, including medical procedures, occupational activities, and accidental or environmental exposures.

Notably, studies conducted by Smith et al. (2003) have shed light on the adverse effects of low-dose radiation. Their research has demonstrated that even at low doses, radiation can induce DNA damage and genomic instability, posing risks to the integrity of genetic materials. These findings underscore the importance of exploring the biological consequences of low-dose radiation exposure and the potential implications for long-term health outcomes. In a comprehensive study conducted by Brenner and Sachs (2006), the risks of radiation-induced cancer at low doses were thoroughly examined. The findings challenge the prevailing assumption that risks are only significant at high doses and raise the possibility of non-linear responses to radiation. Furthermore, a recent meta-analysis (Little et al., 2012), further supports the notion that even low levels of radiation may contribute to long-term health consequences. This meta-analysis synthesized data from multiple occupational cohorts and revealed an increased risk of cancer mortality associated with cumulative low-dose radiation exposure. Such findings highlight the complexity of radiation effects and underscore the importance of investigating low-dose radiation impacts in various contexts.

To comprehensively investigate the potential health consequences of low-dose radiation exposure, researchers are increasingly turning to genome-wide expression data analysis as a powerful tool for uncovering molecular changes and understanding the underlying biological mechanisms. By profiling gene expression patterns across the entire genome, researchers can identify differentially expressed genes, biological pathways, and regulatory network modules that are influenced by low-dose radiation. Currently, gene expression data analysis methods can be broadly categorized into two main categories: hypothesis-driven differential gene expression analysis (Amundson et al., 2003; Jin et al., 2008; Luo et al., 2022) and machine learning approaches (Pirooznia et al., 2008; Park et al., 2019; Cho et al., 2021). Differential gene expression analysis serves the purpose of identifying genes that exhibit differential expression between distinct experimental conditions or groups. This type of method encompasses well-established approaches such as *t*-tests, fold-change analysis, and analysis of variance. These approaches play a pivotal role in elucidating genes that are significantly upregulated or downregulated, thereby shedding light on potential targets that warrant further investigation. On the other hand, the utilization of machine learning techniques has gained significant popularity in the analysis of genome-wide expression data. Supervised machine learning algorithms, including support vector machines (Brown et al., 2000), random forests (Kong and Yu, 2018), and neural networks (Tan and Pan, 2005), have proven instrumental in tasks such as classification and prediction. These algorithms enable the identification of meaningful patterns and relationships within gene expression profiles, facilitating the prediction of biological outcomes or sample classification based on gene expression patterns. Meanwhile, unsupervised learning algorithms, such as self-organizing maps (Tamayo et al., 1999) and Gaussian mixture models (McNicholas and Murphy, 2010), provide valuable assistance in the exploration of hidden patterns or subgroups within the data. These algorithms enable the identification of co-expression modules or clusters, aiding in the discovery of novel biological insights and revealing potential regulatory relationships.

The primary objective of this article is to provide a comparative examination of the effects of low-dose radiation using two distinct analytical approaches: a pathway-based differential gene expression analysis method and a pathway-constrained machine learning method based on deep generative models. The first approach, inspired by the probabilistic pathway activity inference scheme outlined in (Luo et al., 2022; Su et al., 2009), focuses on assessing the activity levels of biological pathways in response to low-dose radiation. This method allows for a comprehensive understanding of the molecular mechanisms and biological processes affected by radiation exposure. The second approach, based on the techniques described in (Seninge et al., 2021a), employs machine learning algorithms to infer and interpret the activity of biological networks in gene expression data. By comparing the results obtained from these two approaches, we aim to gain insights into the impacts of low-dose radiation on gene expression patterns, identify differentially affected pathways, and explore the potential of machine learning in uncovering hidden biological network activity. This comparative analysis will contribute to a better understanding of the molecular consequences of low-dose radiation exposure and provide valuable information for future research in radiation biology and related fields.

## 2 Data

### 2.1 Gene expression omnibus

The data for our study was collected from the Gene Expression Omnibus (GEO) database, a comprehensive archive of gene expression data (Barrett et al., 2012). For our specific focus on low-dose radiation response, we chose the human gene expression dataset GSE43151^1^ from GEO. This dataset includes gene expression profiles from human cell lines subjected to various radiation levels and consists of 121 blood samples from five healthy male donors, each contributing 400 mL of venous peripheral blood Nosel et al. (2013). The samples represent a range of radiation doses, from low to high. Prior to analysis, the GSE43151 dataset underwent a series of preprocessing steps using R GAGE software (Luo et al., 2009). This involved normalizing and filtering the data, resulting in 10,875 probes for further study. We excluded probes undetected in 75% of the samples to ensure data reliability. These steps were essential to reduce data noise and improve the identification of significant biological pathways and their molecular characteristics.

**TABLE 1:** Description of the gene expression dataset GSE43151 that was used to investigate the molecular signatures of low-dose radiation response in this study.

Dose level (Gy)	Number of samples
0	18
0.005	16
0.01	18
0.025	18
0.05	17
0.1	18
0.5	16

### 2.2 Pathway database

We used the KEGG (Kyoto Encyclopedia of Genes and Genomes) database to obtain a reliable set of known biological pathways (Kanehisa and Goto, 2000). KEGG is a collection of manually drawn pathway maps for understanding high-level functions and utilities of biological systems. The genomic information is maintained in the GENES database, which is a collection of gene catalogs for all fully sequenced genomes and some partially sequenced genomes with current annotations of gene functions. The PATHWAY database’s higher-order functional information is augmented with a collection of ortholog group tables for information about conserved subpathways, which are frequently encoded by positionally related genes on the chromosome and are especially valuable in predicting gene functions. In our case, we identified 343 pathways relevant to the gene expression dataset GSE43151 from the available 548 KEGG pathway maps by discarding the pathways that do not contain any gene whose measurement was included in GSE43151.

## 3 Approaches

### 3.1 Approach 1: probabilistic pathway activity inference

To perform the pathway analysis, we first identified the genes whose measurements were included in the gene expression dataset GSE43151 for the pathways of our interest. For every pathway, member genes that were missing in the given dataset were removed from the gene set. Consider a pathway G with *n* genes ${g_{k}}_{k=1}^{n}$ . We assume the expression of gene *g*_*k*_ varies depending on the phenotype. For our analysis, phenotypes were classified based on radiation exposure: zero-dose, low-dose, and high-dose. We evaluated the expression level of gene *g*_*k*_ under each phenotype, assuming Gaussian distribution for simplicity (Luo et al., 2022). The key metric we calculated is the log-likelihood ratio (LLR) for each gene’s expression level, represented by *L*_*k*_(*x*):

$$L_{k}\left(x\right)=\log\left[f_{k}^{1}\left(x\right)/f_{k}^{2}\left(x\right)\right]$$ (1)

The LLR *L*_*k*_(*x*) indicates which phenotype is more likely based on the expression level of gene *g*_*k*_. We aggregated the LLR of all genes in a pathway to assess its overall activity, defining the pathway activity score *S*_*j*_ for sample *j* as:

$$S_{j}=\overset{n}{\underset{k=1}{\sum }}L_{k}\left(x_{j,k}\right)$$ (2)

Given the potential sensitivity of LLR to small data variations, we normalized these scores to ${\hat{L}}_{k}\left(x\right)$ using the following normalization:

$${\hat{L}}_{k}\left(x\right)=\frac{L_{k}\left(x\right)-E\left[L_{k}\left(x\right)\right]}{\sqrt{E\left[{\left(L_{k}\left(x\right)-E\left[L_{k}\left(x\right)\right]\right)}^{2}\right]}}.$$ (3)

While the use of (1, 2) without normalization for inferring the pathway activity level would be equivalent to using a Naive Bayes model (NBM) for classifying the phenotype (class label) given the expression profile of the member genes that belong to a given pathway, this normalization step in (3) makes the pathway activity scoring scheme diverge from the traditional NBM.

To examine the ability of a pathway to discriminate between two phenotypes, we computed the *t*-test statistics scores using the activity levels *S*_*j*_ for all member genes (as defined in (2)) and averaged the absolute value of the *t*-test scores to compute an aggregated differential activity score. The aggregated score–which we refer to as the *pathway activity score*–was then used as an indicator of the pathway’s discriminative power (Tian et al., 2005). It should be noted that low-dose and high-dose samples were analyzed separately to detect the most strongly differentially activated pathways under each radiation exposure level. We had three types of samples: zero radiation, low-dose radiation (0.005 Gy–0.1 Gy), and high-dose radiation (0.5 Gy). Although different low-dose levels of ionizing radiation have been tested, we treated all dose levels between 0.005 Gy and 0.1 Gy as the same type (i.e., low-dose radiation). Based on this categorization, we ranked all relevant KEGG pathways based on the strongest differential pathway activity between zero-dose against low-dose radiations, and separately, based on zero-dose against high-dose radiations.

### 3.2 Approach 2: pathway-constrained gene expression analysis using VEGA

Lately, the emergence of deep generative models such as variational autoencoders (VAEs) has facilitated the understanding of cellular mechanistic responses under different perturbations based on gene expression profiles (Lopez et al., 2018; Niyakan et al., 2021). These deep models have the potential to capture high-order nonlinear bio-molecular interactions; however, one of their main limitations is the lack of interpretability for the latent space that these models infer. By incorporating prior biological knowledge such as gene pathway information, pathway-constrained VAE-based models, for example, VEGA–VAE Enhanced by Gene Annotations (Seninge et al., 2021b), can provide interpretability for learned latent variables as inferred pathway activity scores.

More specifically, in the VEGA architecture, the decoder is a sparse single-layer neural network whose neuron connections are mirroring the user-provided gene-pathway maps, while the encoder embeds the input gene expression profiles into the latent space through a nonlinear neural network. The generative part of VEGA for reconstructing gene expression is designed to be a masked linear decoder, in which each latent variable (pathway) is directly connected to an output gene if this gene is previously annotated to be a member of this pathway. This choice of the decoder architecture in VEGA enforces the encoding of the prior biological knowledge that genes work together in coordination in pathways while the deep neural network encoder and decoder capture nonlinear high-order interactions. Here, we use the pathway prior knowledge previously described in Section 2.2, and thus the decoder wirings are based on the corresponding gene-pathway mapping relationships.

In this article, for training the VEGA models in the experiments, we have taken a learning rate of 1*e* − 4 with the Adam optimizer. We have trained all the models for a maximum of 200 epochs to avoid overfitting, with early stopping implemented in VEGA to stop the training procedure when the training or validation loss stops decreasing for 10 consecutive epochs. For the encoder and decoder architectures, we have followed the instructions in the original VEGA paper (Seninge et al., 2021b): The encoder is composed of two layers of fully connected nodes with the input number of features being the same as the number of genes in the gene expression dataset and the number of its first layer output features set to be 800. The latent space dimension is set to be the number of extracted pathways from the KEGG database (343 pathways as described in Sec. 2.2) plus one additional fully connected node to capture additional data variability, which leads to a latent space dimension of 344 in the trained VEGA models. The decoder is a sparse single-layer neural network as described previously to reconstruct gene expression based on the pathway-constrained latent representations.

Differential pathway activities are often of interest when contrasting two different groups of cells. Inspired by the Bayesian hypothesis testing procedure from Lopez et al. (2018), for the differential pathway activity analysis, the posterior probabilities of mutually exclusive hypotheses are approximated through repeated Monte Carlo sampling of the correspondingly derived VEGA’s latent variable distributions. Then, the pathways are ranked by estimated Bayes factor (Held and Ott, 2018), the ratio of the hypothesis posteriors. The sign of the corresponding Bayes factor indicates which of the null and alternative hypotheses is more likely, and its magnitude represents the significance level of the pathway differential activity.

## 4 Results

### 4.1 Threshold for categorizing radiation doses

Defining low and high radiation doses remains a debated topic in scientific literature and regulatory standards. The National Council on Radiation Protection and Measurements (NCRP) suggests low doses are typically below 0.1 Gy, while high doses exceed 1 Gy (Wood, 1994). Our study’s gene expression data ranges from 0.005 Gy to 0.5 Gy, fitting within the NCRP’s low-dose category. We analyzed pathway activities across these doses to understand their differential effects.

**FIGURE 1:**
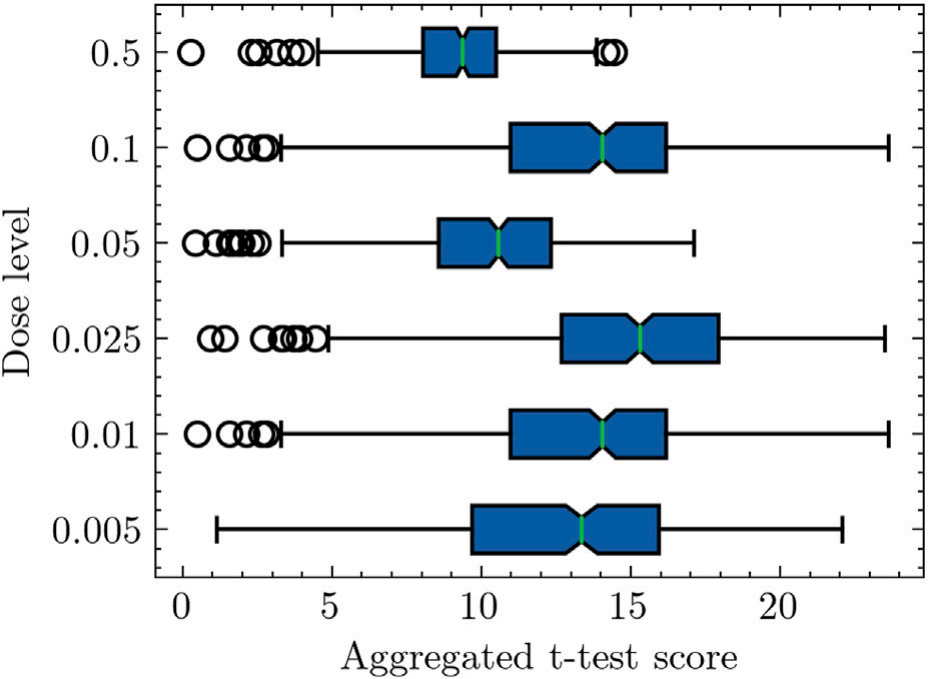
Box plot representation of pathway activity scores across radiation doses. The pathway activity score computation involves a two-step process. In the first step, the aggregated log-likelihood ratio (LLR) is calculated for each pathway. For instance, considering a specific pathway comprising 20 genes, the aggregated LLRs are determined using Eq. 2. Subsequently, the second step involves the calculation of the pathway activity score. In this context, for a radiation dose level of 0.005, corresponding to 16 samples (refer to Table 1), the pathway activity score is derived by averaging the absolute *t*-test scores of the aggregated LLRs across these 16 samples.

Figure 1 presents a box plot representation, where the *x*-axis represents the aggregated differential activity score. In our case, it is aggregated *t*-test score, which is acquired by averaging the absolute *t*-test scores of individual genes within a specific pathway. On the other hand, the *y*-axis represents the radiation dose level. The findings from the plot unveil different patterns in the computed pathway activity scores across various radiation doses. First, the box plot illustrates that samples exposed to a radiation dose of 0.5 Gy exhibit the lowest pathway activity scores among all the considered doses. Similarly, samples exposed to a dose of 0.05 Gy display relatively lower pathway activity scores, implying a comparatively milder impact on gene expression when compared to other low doses. In contrast, samples exposed to different radiation dosages demonstrate considerably higher pathway activity scores, indicating greater differential separability in terms of gene expression profiles. Moreover, the plot reveals a trend as the radiation dose increases from 0.005 Gy to 0.025 Gy. The computed pathway activity scores mean progressively rise, indicating an overall shift towards higher pathway activity scores. This observation suggests that as the radiation dose escalates within this range, the differential activities in gene expression become more pronounced.

**FIGURE 2:**
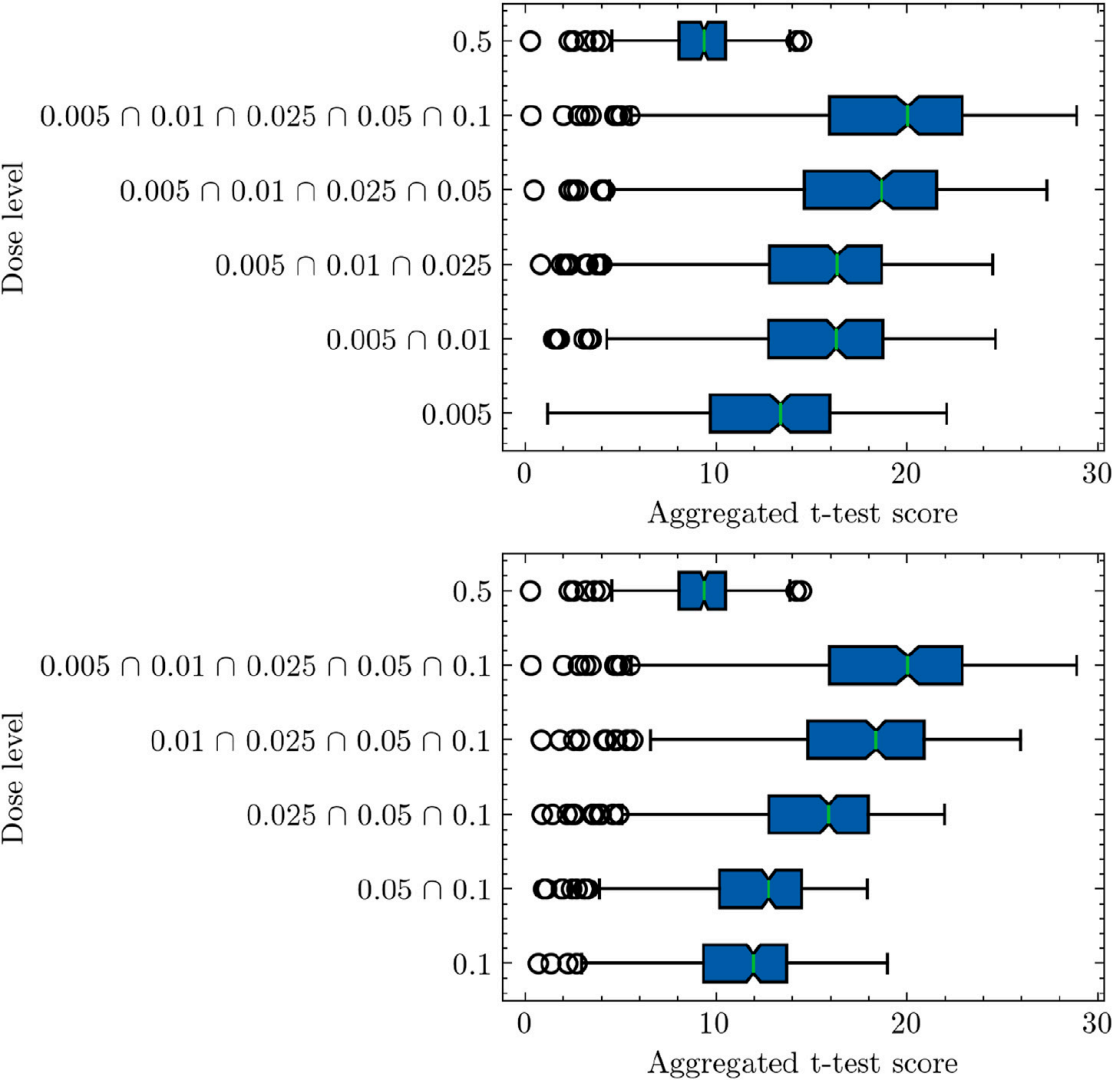
Pathway-based analysis of log-likelihood ratios (LLRs) for low-dose and high-dose samples, supporting the threshold of 0.1 Gy as a distinguishing criterion. The difference observed in the analysis outcomes depicted in Figures 1, 2 arises from their respective experiment settings. Figure 1 shows individual pathway activity scores associated with each discrete dose level. In contrast, Figure 2 presents pathway activity scores that commence from the lowest dose level, progressively incorporating additional low-dose samples in an ascending sequence, as indicated by the annotations on the *y*-axis labels. This progressive inclusion approach offers a nuanced perspective on the pathway activity trends across the spectrum of low doses.

Figure 2 provides an in-depth analysis of the pathway-based differential activity for samples categorized as low-dose or high-dose, following the establishment of a threshold of 0.1 Gy. The experiment follows a sequential approach, starting from the lowest dose of 0.005 Gy and progressively incorporating additional low doses in ascending order. Recall that the pathway activity score computation utilizes a single-sample *t*-test approach. In this context, the null hypothesis posits that the pathway activation score maintains a mean of 0, thereby lacking informative content regarding the data. Conversely, the alternative hypothesis suggests a nonzero mean for the activity test, implying positive values for Low samples and negative values for zero samples. Consequently, the computation involves evaluating the ratios *p*_1_(*x*)/*p*_2_(*x*) when *x* originates from class 1, and conversely, *p*_2_(*x*)/*p*_1_(*x*) when *x* originates from class 2. In Figure 2, as more samples from various low dosages are included in the analysis, the computed pathway activity scores demonstrate a consistent upward trend. Note a higher *t*-statistic corresponds to an increased propensity for rejecting the null hypothesis. With the progressive inclusion of an expanded set of low-dose samples achieved through the combination of diverse low doses, the computed t-statistic exhibits a notable increase in magnitude. This phenomenon serves to strengthen the evidential basis that supports the rejection of the null hypothesis in favor of the alternative hypothesis. This finding provides empirical support for the selection of 0.1 Gy as a reasonable threshold to distinguish between low-dose and high-dose radiation in our study. Meanwhile, despite the limited quantity of data available, the shift in the computed LLR distribution consistently follows a monotonic pattern. This suggests that the adopted pathway-based differential activity analysis can effectively capture the dose-dependent effects on gene expression (Luo et al., 2022). A potential issue with adding more low-dose data is the increase in sample size and statistical power, potentially leading to higher *t*-test scores without a change in effect size. To address this, we conducted an analysis in reverse order, starting with 0.1 Gy, then including 0.1 and 0.05 Gy, and continuing in this manner. This reverse-order analysis confirmed consistency with our initial ascending-order findings, supporting the robustness of our study’s conclusions.

### 4.2 Integration of low-dose data

We would also like to highlight the uniqueness of samples across different radiation dosage levels in our study. Despite using a threshold of 0.1 to distinguish between low-dose and high-dose samples, we still have five distinct dosage levels falling within the low-dose category. It is important to note that due to the limited number of samples, which is a common scenario in biological experiments, the results of the pathway-based differential activity analysis can vary among different dosages. To provide a comprehensive overview, Table 2 presents the top five pathways ranked based on the calculated LLRs for each dose. It is worth mentioning that the analysis was performed independently for each dosage level, and the low-dose samples are divided into subgroups based on their respective dosages. Based on the findings presented in Table 2, there is no overlap observed among the top five ranked pathways. Each of the identified pathways in the low-dose and high-dose categories appears to be distinct and unique, without any shared representation within the top five rankings.

**TABLE 2:** Top five ranked pathways for each radiation dosage level. The color scheme used in the table distinguishes between low-dose (represented by the color blue) and high-dose (represented by the color red). The pathway name can be retrieved by searching for the entry ID number at https://www.genome.jp/kegg/kegg2.html.

Rank	0.005 Gy	0.01 Gy	0.025 Gy	0.05 Gy	0.1 Gy	0.5 Gy
*#*1	hsa05167	hsa05131	hsa04714	hsa05110	hsa05146	hsa05202
*#*2	hsa05170	hsa05130	hsa04723	hsa04015	hsa05222	hsa04110
*#*3	hsa04144	hsa04120	hsa05415	hsa05012	hsa04120	hsa04310
*#*4	hsa04120	hsa04022	hsa05166	hsa04966	hsa05212	hsa05203
*#*5	hsa05022	hsa04922	hsa01100	hsa00410	hsa05131	hsa04390

**FIGURE 3:**
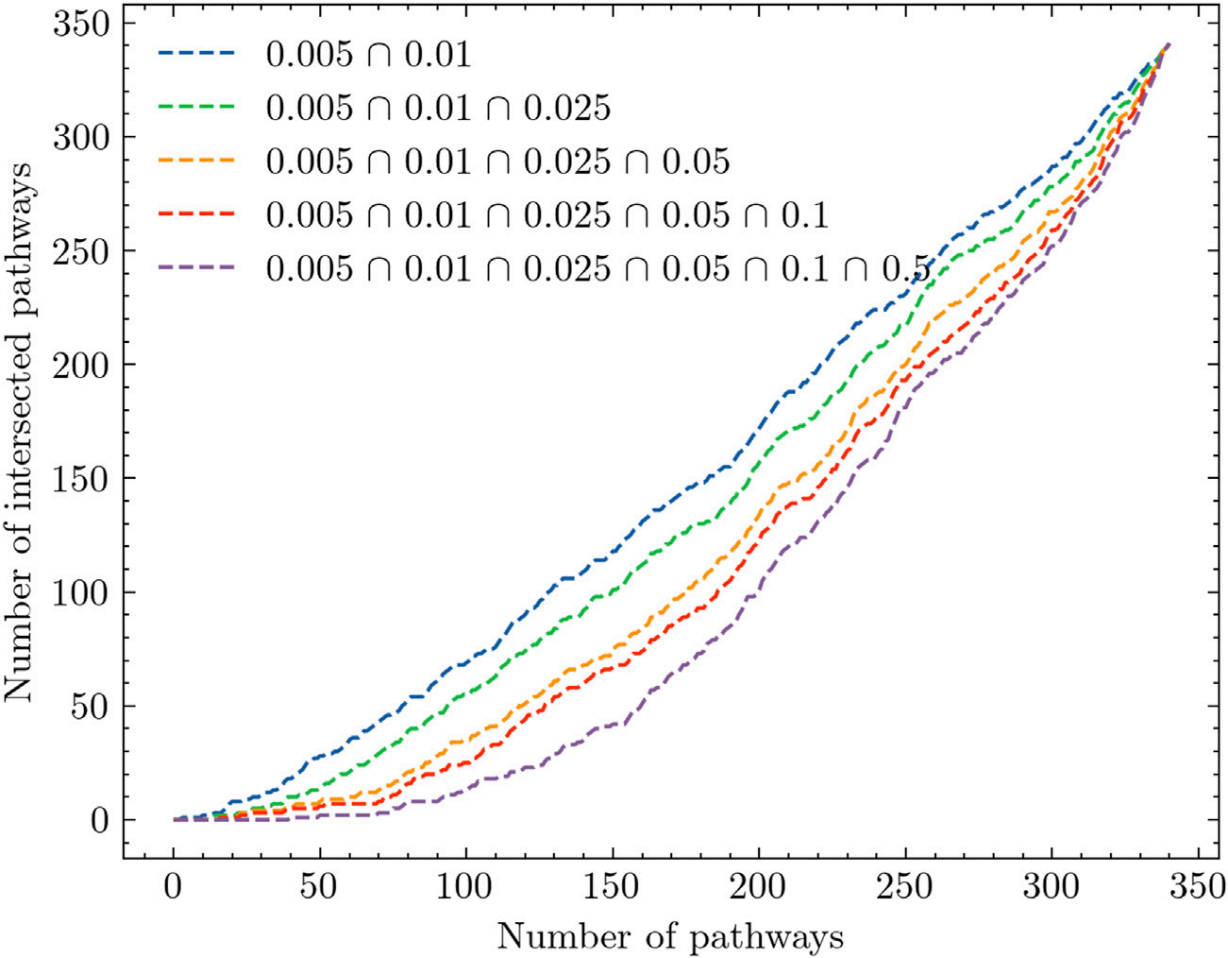
Analysis of pathway intersection across different radiation dose levels.

Figure 3 presents an extended analysis of the results. The *x*-axis of Figure 3 represents the number of KEGG pathways, while the *y*-axis represents the number of intersected pathways observed across the experiments. Each experiment corresponds to a specific radiation dosage level, and the pathways are ranked accordingly. The figure comprises a line plot that illustrates the list intersection of the pathways. For example, the blue line in the plot is obtained by iteratively counting the elements that are common to both the 0.005 Gy and 0.01 Gy experiments. This line represents the intersection of pathways observed between these two dosage levels. By comparing the different lines, we can observe that as we include more experiments to identify the number of overlapped pathways, the slope of the line gradually decreases. This suggests that as the number of experiments increases, the extent of pathway intersection diminishes. In particular, without distinguishing between low-dose and high-dose samples, we find that there are no overlapped pathways among the top 50 ranked pathways (See the purple line). This finding provides further evidence for the intrinsic complexity pattern inherent in the gene expression data itself.

**FIGURE 4:**
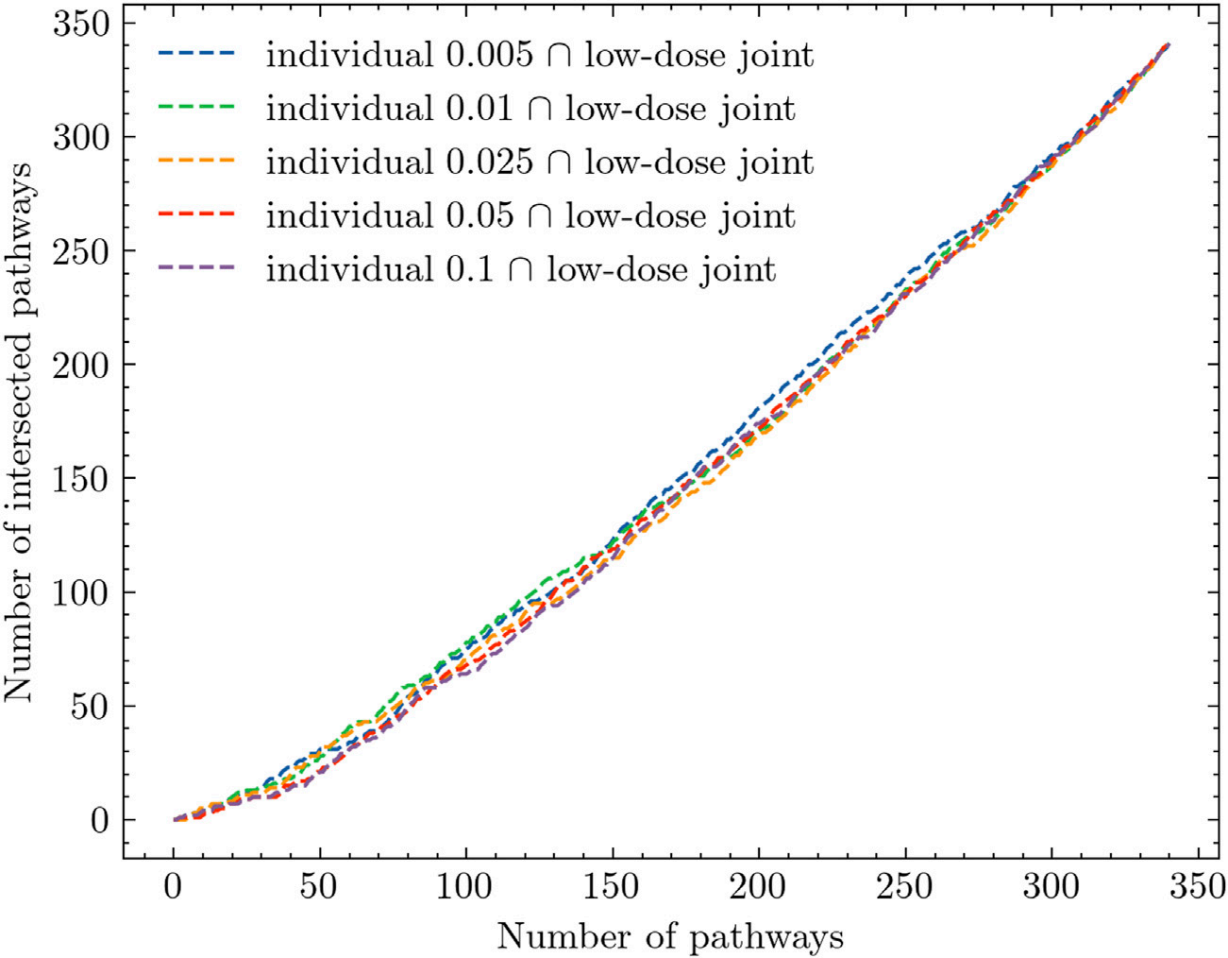
Intersection analysis of individual experiments and low-dose joint analysis.

Based on the analysis results presented in Table 2; Figure 3, there is evidence to suggest that combining all low doses and conducting a joint analysis, referred to as the low-dose joint analysis, would yield better insights. To validate this assumption, we performed the same intersection analysis between each experiment and the low-dose joint experiment. The results of this analysis are presented in Figure 4. In Figure 4, we observe a distinct difference compared to Figure 3. The low-dose joint analysis successfully integrates information from different doses, and the intersections between different doses and the low-dose joint experiment exhibit a similar pattern. This suggests that by combining the low-dose samples, we can capture common pathway interactions that are shared among different low-dose samples. Within the top 50 pathways, we observe a notable number of pathways that are overlapped between different comparison experiments and the low-dose joint experiment. This finding indicates that the integration of low-dose samples enhances the identification of shared pathways across various radiation dose levels. By pooling the low-dose data together, we can capture common molecular mechanisms and gain a more comprehensive understanding of the underlying biological processes affected by radiation exposure.

### 4.3 Pathway-based differential activity analysis results

**FIGURE 5:**
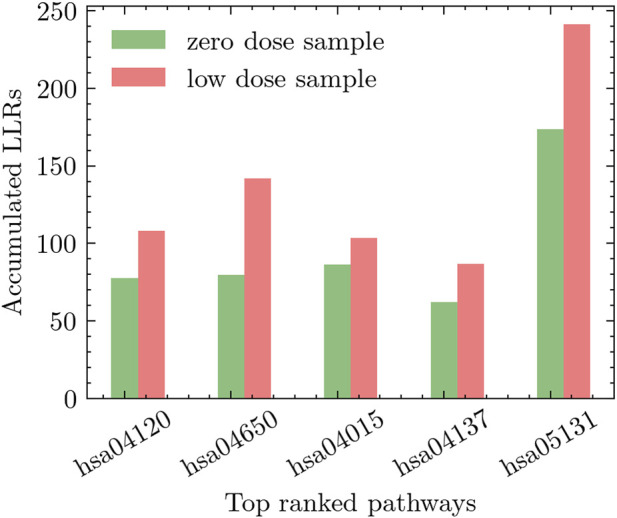
Top five pathways showing significant differential activation in response to low-dose radiation compared to zero-dose.

**FIGURE 6:**
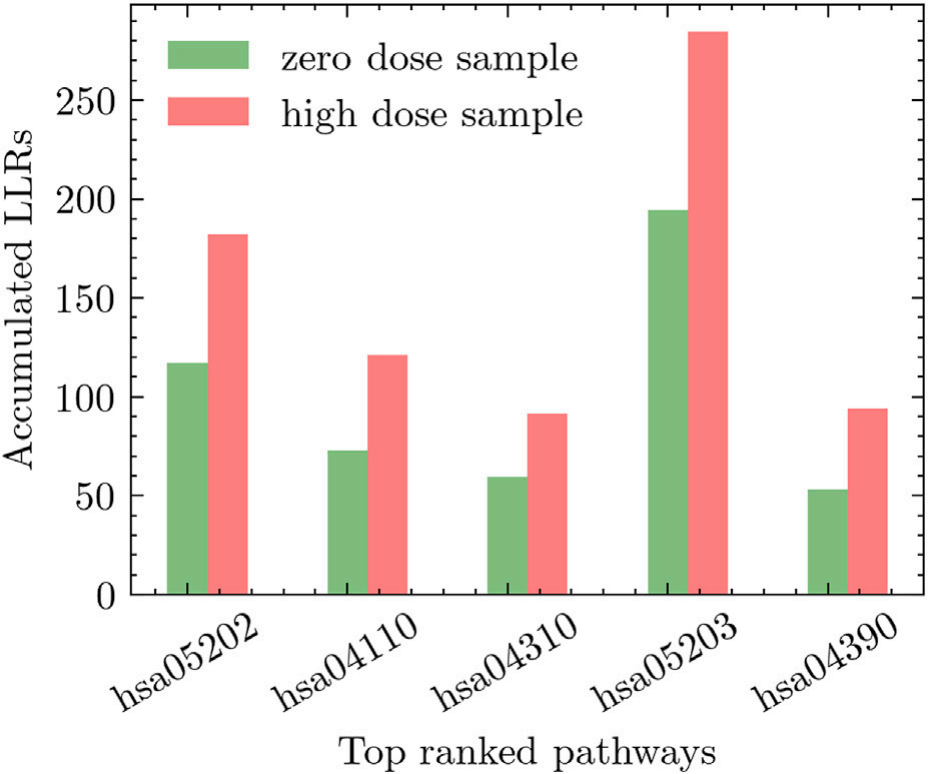
Top five pathways showing significant differential activation in response to high-dose radiation compared to zero-dose.

We conducted two distinct experiments to explore the effects of radiation exposure. The first experiment compared *high-dose* radiation (0.5 Gy) to *zero-dose*, while the second experiment focused on comparing *low-dose* radiation (including all low-dose samples) to *zero-dose*. To assess the impact of radiation on different pathways, we performed an extensive evaluation of relevant pathways in the KEGG database. The pathways were ranked based on their discriminative power using the methodology described in Section 3.1. This ranking approach considered the accumulated differential activity score, which was computed by averaging the absolute values of the *t*-test scores of the genes within each pathway and estimating the corresponding *p*-values. In Figure 5, we present the top five pathways that exhibited the most significant differential activation in response to low-dose radiation. Similarly, Figure 6 showcases the top five pathways that demonstrated the highest degree of differential activation under high-dose radiation conditions.

The *hsa04120-Ubiquitin mediated proteolysis* pathway emerges as the top pathway in the experiment of low-dose radiation. Accumulating evidence suggests that low-dose radiation has the ability to modulate this pathway, resulting in the degradation of specific proteins involved in essential cellular processes, including cell cycle regulation and DNA repair. For instance, Zheng et al. (2019) provides insights into the impact of low-dose radiation on the Ubiquitin-mediated proteolysis pathway. It is observed that exposure to low-dose radiation selectively triggered the degradation of specific cell cycle regulators, such as cyclin-dependent kinase inhibitors, through the ubiquitin-proteasome pathway. Similarly, the work in He et al. (2019) shed further light on the link between low-dose radiation and the Ubiquitin-mediated proteolysis pathway. Their study demonstrated that low-dose radiation exposure can activate this pathway, resulting in the degradation of specific proteins involved in DNA repair, cell cycle regulation, and apoptosis.

In experiments involving high-dose radiation, the hsa05202-Transcriptional misregulation in cancer pathway was notably prominent. Research has shown that high-dose radiation affects various cellular processes at the transcriptional level, particularly those linked to cancer. It was observed that this pathway plays a significant role in critical cellular functions such as proliferation, survival, cycle progression, and apoptosis. These processes are particularly vital in understanding the cellular alterations induced by high-dose radiation, illustrating the pathway’s impact on cellular dynamics in such conditions (Choudhary et al., 2020; Wei et al., 2021).

**FIGURE 7:**
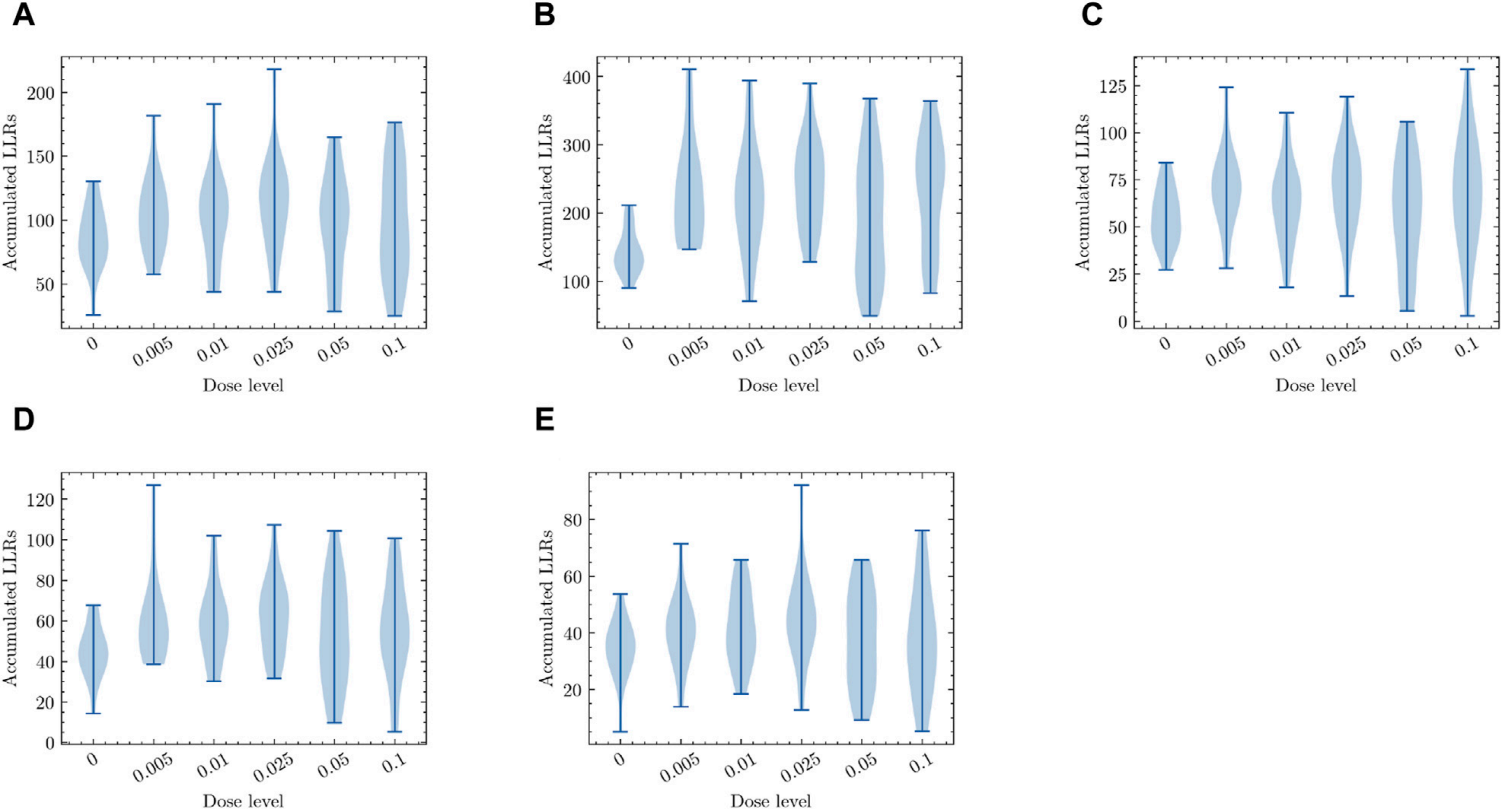
The pathway activity level measured in terms of the accumulated log-likelihood ratios (LLRs) in response to different levels of radiation exposure. Dose-dependent activity level is shown for the top five pathways that were most differentially activated under low-dose radiation exposure. **(A)** Ubiquitin mediated proteolysis **(B)** Natural killer cell mediated cytotoxicity **(C)** Rap1 signaling pathway **(D)** Mitophagy **(E)** Shigellosis. All plots in **(A–E)** for the top low-dose response pathways display similar trends, where the differential activity levels reflecting the presence of potential molecular signatures of low-dose radiation response decrease as the radiation dose level increases.

Next, we examined the effects of different radiation dosages on the top-ranked pathways that are highly sensitive to low-dose radiation exposure. As discussed earlier in Section 3.1, we employed a probabilistic pathway activity inference scheme (Su et al., 2009), which can be seen as a simplified probabilistic graphical model (PGM), specifically an NBM (Naive Bayes Model). Equation 2 was used to calculate the pathway activity score based on the log-likelihood ratios (LLRs) of the individual genes within the pathway. Our objective was to determine whether this PGM, designed to detect the presence of low-dose radiation exposure, consistently produces reliable activity inference results when the radiation dosage varies. Figure 7 illustrates the inference results obtained from the PGM trained to differentiate between *zero-dose* and *low-dose* samples. The *y*-axis represents the aggregated LLRs, while the *x*-axis represents the radiation dose levels. To visualize the data distributions, we employed violin plots for each dosage level, which show the range, median, and distribution of the accumulated LLRs. The results focus on the top five pathways that exhibited the highest responsiveness to low-dose radiation. As depicted in Figure 7, all these pathways displayed similar trends, with the inferred differential activity levels generally decreasing as the radiation exposure level increased. Due to the limited number of available samples for analysis, the violin plots show a wide distribution range. However, the mean and median values provide a clear indication of the observed trend. These findings suggest that these pathways, along with the gene expression profiles of their constituent members, may serve as potential molecular signatures associated with the biological response to low-dose radiation exposure.

### 4.4 Pathway analysis results using VEGA

To decipher the response differences with different radiation exposure levels, we further discuss the pathway activity inference results of applying VEGA, which may help identify nonlinear high-order molecular interactions as previously described in Section 3.2. The gene expression data were pre-processed by following the same steps noted in Section 2.1. We have separated the normalized gene expression data into two different sets of samples based on radiation levels explained in Section 4.1: 1) Samples with zero- and low-dose exposure; and 2) Samples with zero- and high-dose radiation exposure. For each of these two different groups of samples, a VEGA model has been trained for 200 epochs to embed the gene expression data into the lower-dimensional interpretable pathway-constrained latent space to infer the pathway activity.

**FIGURE 8:**
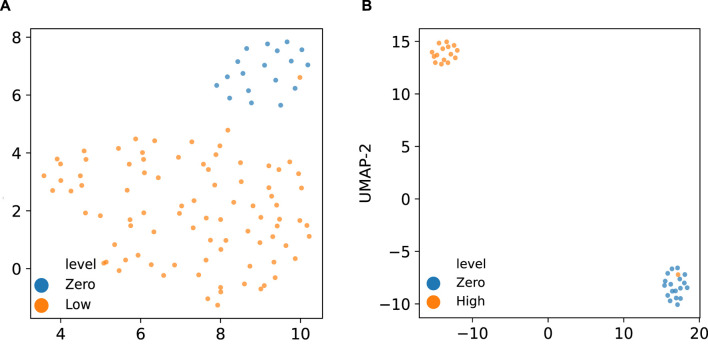
UMAP embedding of the latent space inferred by the VEGA models trained on **(A)** zero and low-dose, and **(B)** zero and high-dose radiated samples colored by their corresponding radiation exposure levels.

Figure 8 displays the UMAP (Uniform Manifold Approximation and Projection) (McInnes et al., 2020) embedding of the derived latent space by the corresponding VEGA models when trained on the two groups of gene expression samples. As indicated in these visualized embeddings in Figure 8, the latent space learned by VEGA has captured the transcriptomic response to different radiation levels as embedded points are clustered together based on their corresponding radiation exposure in both combinations of the (zero,low)-dose and (zero, high)-dose sample groups. While there are differential pathway activities clearly distinguishing zero- and high-dose samples, differential activities between zero- and low-dose samples can be more subtle and require careful investigation.

To identify the KEGG pathways that are differentially activated in either low-dose or high-dose radiation exposure, compared to samples with no radiation exposure, we applied the Bayesian hypothesis testing procedure that has been implemented in VEGA as described in Section 3.2 on both the (zero,low)-dose and (zero, high)-dose sample groups.

**FIGURE 9:**
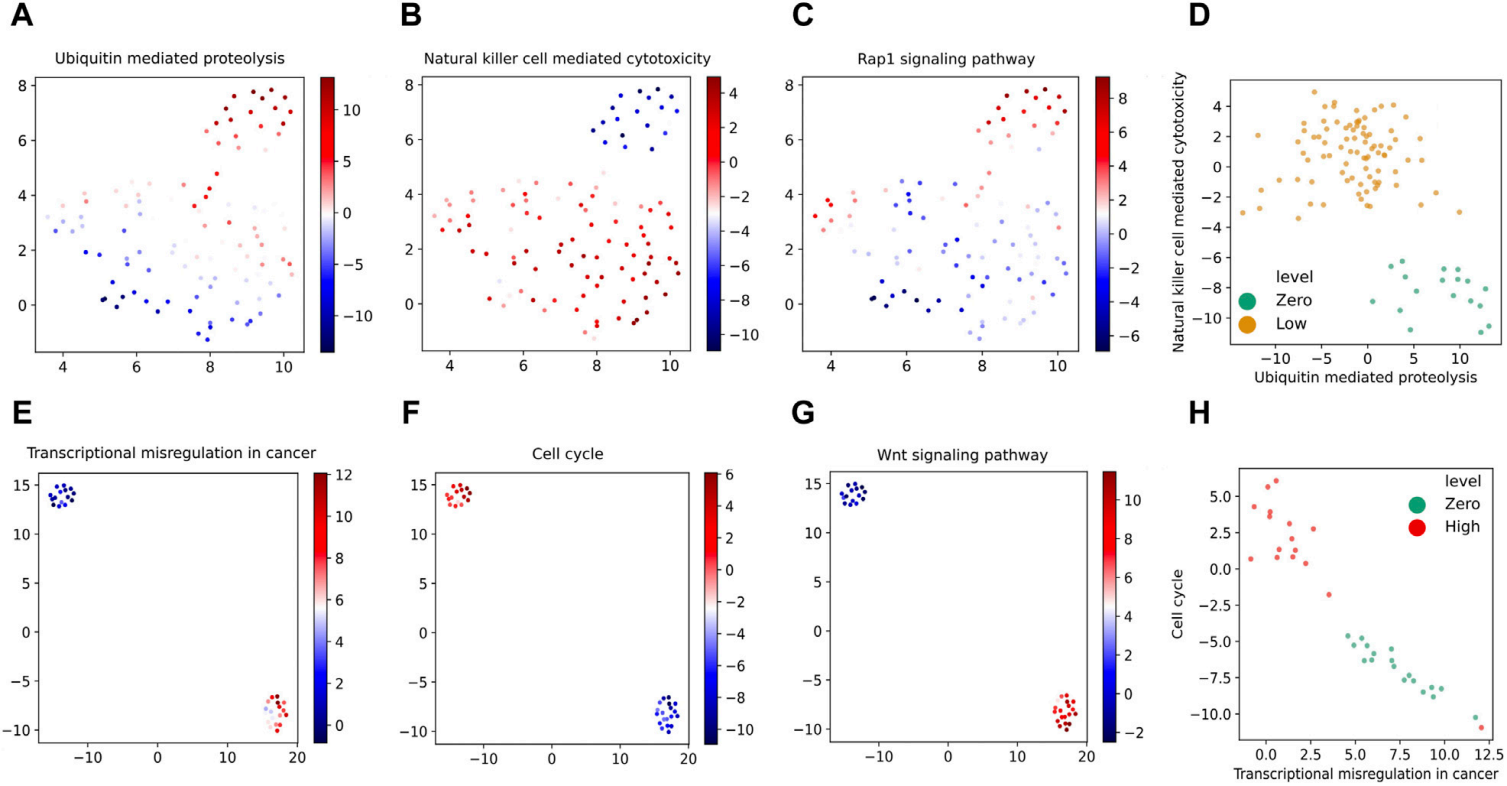
UMAP embedding of the latent space inferred by the VEGA models with the samples colored according to the VEGA-inferred activities of our identified top three most differentially activated KEGG pathways when trained on zero- and low-dose samples **(A–C)** and zero- and high-dose samples **(E–G)**. Bivariate VEGA-inferred pathway activity scores plots of the top two KEGG pathways resulting from our differential pathway activity analysis on (zero,low) and (zero, high) dose samples are shown in **(D,H)** respectively.

Figures 9A–C show the same UMAP embedding plots of the corresponding VEGA latent space when trained on zero- and low-dose samples as shown in Figure 8A with the samples now colored according to the VEGA-inferred activities of *Ubiquitin mediated proteolysis*, *Natural killer cell mediated cytotoxicity* and *Rap1 signaling pathway* KEGG pathways, respectively. These are the top three pathways that we identified as the most differentially activated in the presence of low-dose radiation and discussed them in detail in Section 4.3. These pathways as shown in these plots have differential VEGA-derived pathway activity scores between low- and zero-dose samples. To quantify the pathway’s differential activity between low- and zero-dose samples, we have calculated the Bayes factors for each of the KEGG pathways as we described previously. The *Natural killer cell mediated cytotoxicity* pathway that was previously identified as the second top differentially activated pathway in the presence of low-dose radiation by our proposed pathway-based analysis, is also ranked as the top differentially activated pathway in the trained VEGA model by having the log_*e*_(|BF|) of 27.6. The calculated log_*e*_(|BF|) for the *Ubiquitin mediated proteolysis* and *Rap1 signaling pathway* KEGG pathways was 3 and 2.6, respectively. There is strong evidence for the differential activation of these pathways with log_*e*_(|BF|) greater than 2.3 (equivalent to having |BF| > = 10) in the Bayesian hypothesis testing framework. Additionally, we have observed that low- and zero-dose samples are well-segregated in the two-dimensional VEGA-based pathway activity score visualization of the top two KEGG pathways resulting from our differential pathway activity analysis as shown in Figure 9D. Overall, these VEGA-based pathway analysis results confirm our previous findings derived by probabilistic pathway-based analysis of gene expression profiles in low-dose radiation exposure.

We further perform VEGA-based pathway analysis for the (zero, high)-dose group of samples. By visualizing the UMAP embedding plots of the VEGA latent space trained with zero- and high-dose samples as shown in Figure 8B with the samples colored according to the corresponding VEGA-inferred activities of *Transcriptional misregulation in cancer*, *Cell cycle* and *Wnt signaling pathway* KEGG pathways, we can observe their differential activity as depicted in Figures 9E–G respectively. We have previously identified these three pathways as the top three differentially activated KEGG pathways in the presence of high-dose radiation as shown in Figure 6. Same as what we have done for the (zero,low)-dose sample groups, we further calculate the Bayes factors for these KEGG pathways by following the same statistical hypothesis testing procedure implemented in VEGA. The calculated log_*e*_(|BF|) values for the *Transcriptional misregulation in cancer*, *Cell cycle* and *Wnt signaling pathway* pathways are 2.8, 2.8 and 3. As the Bayes factor values suggest, these three pathways are also detected to be differentially activated in the presence of high-dose radiation by VEGA as well since they all have log_*e*_(|BF|) higher than the significance level threshold of 2.3. Moreover, in Figure 9H where the samples (colored by their radiation exposure levels) are plotted according to the bivariate VEGA pathway activity scores of the top two KEGG pathways resulting from our differential pathway activity analysis, we can see that the zero- and high-dose samples are separated clearly. This again demonstrates the discriminative power of the top most differentially activated pathways that we identified previously.

It is worth mentioning that VEGA as a deep generative model has the potential to capture high-order nonlinear interactions in differential pathway activities that might be missed in a pathway-based analysis using Eq. 2 due to the simplifying assumptions made therein. For example, the pathways *Cushing syndrome* and *Riboflavin metabolism* are among top five differentially activated pathways when comparing (zero,low)- and (zero, high)-dose samples by VEGA, with 27.6 and 4.5 log_*e*_(|BF|) values respectively, had relatively small pathway activity scores by Eq. 2 and were not among differentially activated pathways by the first approach. Several studies have discussed and reported the effectiveness of low-dose radiotherapy in the treatment of patients with Cushing’s disease (Ahmed et al., 1984; Mahmoud-Ahmed and Suh, 2002); confirming VEGA-inferred pathway differential activity of *Cushing syndrome* pathway (log_*e*_(BF) = −27.6) in (zero,low)-dose differential activity analysis. These results are indicative of VEGA’s capability in unraveling more complex non-linear interactions inherent in gene expression data as well as the different modeling perspectives of the two pathway-based analysis approaches studied in this work.

In summary, VEGA-based pathway analysis confirms the top differentially activated pathways derived from the statistical pathway-based analysis of gene expression profiles based on the probabilistic graphical model. It also has the potential of capturing additional differentially activated pathways considering non-linear interactions.

## 5 Conclusion

In this study, we conducted a comparative analysis aiming to investigate the response to low-dose radiation exposure and identify potential molecular mechanisms involved. To achieve this, we first performed a pathway-based gene expression analysis. Utilizing a probabilistic pathway activity inference scheme, we assessed the differential activity levels of specific pathways under varying degrees of radiation exposure. Gene expression patterns under the radiation exposure at six different dose levels ranging from 5 mGy to 500 mGy were investigated, where the measurements in the original study (Nosel et al., 2013) were made using blood samples obtained from five different donors during five independent irradiation sessions. This method involved aggregating the log-likelihood ratios (LLRs) of member genes within each pathway to infer their differential activity. By employing this approach, we were able to accurately detect pathways where member genes displayed subtle yet consistent coordinated expression patterns in response to low-dose radiation exposure. To prioritize the pathways, we conducted an extensive search through the KEGG database, focusing on their differential activity levels influenced by low-dose radiation exposure. Through this comprehensive analysis, we successfully identified the top pathways potentially associated with the response to low-dose radiation. We have also performed additional analyses leveraging the pathway-constrained deep neural network model, VEGA, where the comparative analysis also confirms the detected differential pathway activities based on pathway activity scores via aggregated LLRs of member genes. Findings in this study reflect the complicated nature of the biological response to low-dose ionizing radiation, as well as the fact that low-dose exposures affect many different gene pathways that are not significantly altered after higher doses of radiotherapy.

To further enhance our understanding of pathway-based responses to different perturbations, pathway-constrained models that can infer aggregated activity scores capturing nonlinear interactions will be further developed incorporating the perturbation labels and conditions as supervised models to better study coordinated transcriptomic responses to different radiation exposure conditions (Niyakan et al., 2024). Another intriguing avenue for future investigation involves leveraging large language models to extract knowledge about protein interactions, pathways, and gene regulatory relationships from relevant scientific literature (Park et al., 2023b; Park et al., 2023a) and integrating them into the analysis. This has the advantage of detecting and utilizing context-specific molecular interactions (or other relevant prior scientific knowledge) for integrative analysis of transcriptomic data–instead of restricting the analysis to known pathways, which are static (i.e., not context-specific) and potentially incomplete. By incorporating such “context-specific” knowledge extracted by LLMs as priors, we may significantly advance our comprehension of the molecular signatures underlying the cellular response to low-dose (as well as high-dose) ionizing radiation exposure.

## Data Availability

Publicly available datasets were analyzed in this study. This data can be found here: https://www.ncbi.nlm.nih.gov/geo/query/acc.cgi?acc&equals;GSE43151.

## Footnotes

1
https://www.ncbi.nlm.nih.gov/geo/query/acc.cgi?acc=GSE43151
