# Supplementary material for: Pathway-based analyses of gene expression profiles at low doses of ionizing radiation
Source: Front Bioinform. 2024 May 14;4:1280971. doi: 10.3389/fbinf.2024.1280971 (PMC11135168; doi:10.3389/fbinf.2024.1280971)
Supplement: Supplementary file 1 [file Table1.pdf]

Supplementary Material

APPENDIX 1

The table presented below details the KEGG IDs along with the respective names of all pathways identified in our study.

KEGG ID	Pathway Name	T-Statistic Score
hsa04120	Ubiquitin mediated proteolysis - Homo sapiens (human)	28.89025
hsa04650	Natural killer cell mediated cytotoxicity - Homo sapiens (human)	28.12969
hsa04015	Rap1 signaling pathway - Homo sapiens (human)	27.93452
hsa04137	Mitophagy - animal - Homo sapiens (human)	27.03721
hsa05131	Shigellosis - Homo sapiens (human)	26.87964

Table S1. The top five pathways that exhibit notable differential activation when exposed to low-dose radiation, as opposed to zero-dose, are depicted in Figure 5.

KEGG ID	Pathway Name	T-Statistic Score
hsa05202	Transcriptional misregulation in cancer - Homo sapiens (human)	14.43176
hsa04110	Cell cycle - Homo sapiens (human)	14.18099
hsa04310	Wnt signaling pathway - Homo sapiens (human)	13.86136
hsa05203	Viral carcinogenesis - Homo sapiens (human)	13.44048
hsa04390	Hippo signaling pathway - Homo sapiens (human)	13.33797

Table S2. The top five pathways that exhibit notable differential activation when exposed to high-dose radiation, as opposed to zero-dose, are depicted in Figure 6.
